# N-Butyl Cyanoacrylate Is Very Effective for Massive Haemorrhage during the Perinatal Period

**DOI:** 10.1371/journal.pone.0077494

**Published:** 2013-10-11

**Authors:** Suguru Igarashi, Shinichirou Izuchi, Yukihisa Ogawa, Misako Yoshimathu, Kenji Takizawa, Yasuo Nakajima, Mamoru Tanaka, Bunpei Ishizuka, Nao Suzuki

**Affiliations:** 1 Departments of Obstetrics and Gynecology, St. Marianna University School of Medicine, Kawasaki, Japan; 2 Rose Ladies Clinic, Tokyo, Japan; 3 Department of Radiology, St. Marianna University School of Medicine, Kawasaki, Japan; 4 Department of Advanced Reproductive Medicine, St. Marianna University School of Medicine, Kawasaki, Japan; Baylor College of Medicine, United States of America

## Abstract

**Objective:**

The liquid embolic agent n-butyl cyanoacrylate (NBCA) is a tissue adhesive used as an immediate and permanent embolic agent when mixed with oil-based contrast medium. In this study, the preservation of fertility with TAE using NBCA for massive haemorrhage during pregnancy or the peripartum period and the utility of this therapy were investigated.

**Methods:**

Cases from January 2005 to October 2010 in which TAE was performed for massive haemorrhage in pregnant women, particularly during the peripartum period, were investigated.

**Results:**

TAE was performed in 27 pregnant women. The embolic agent used was GS only in five cases, NBCA only in 19 cases, and additional embolization with NBCA when the effect with GS was insufficient in three cases, one each of abruptio placentae, cervical pregnancy, and uterine atony.A comparison of mean blood loss when each embolic agent was used for haemostasis showed a significant difference between cases in which GS only was used and cases in which NBCA only was used. In a comparison of mean transfusion volume, a significant difference was seen between cases in which both GS and NBCA were used and cases in which NBCA only was used. In a postoperative follow-up survey, menses resumed in eight patients, including four patients who later became pregnant and three who delivered.

**Conclusions:**

TAE with NBCA, which has an embolic effect unrelated to clotting dysfunction for massive haemorrhage during the peripartum period, is a minimally invasive and very effective treatment method for patients with severe DIC.

## Introduction

Perinatal bleeding is a leading cause of death in pregnant and parturient women. Causes include abruptio placentae, atonic haemorrhage, birth canal laceration, uterine rupture, and placental defects (e.g., placenta praevia, placenta accreta). In cases of sudden intrapartum bleeding, attempts are first made to stop the bleeding with massage of the uterine fundus, administration of uterotonic agents, and suture of the laceration site. However, if there is excessive blood loss over a short time, haemorrhagic shock or disseminated intravascular coagulation (DIC) may occur, and massive transfusion volumes become necessary. In some cases, total hysterectomy is needed. In recent years, favourable haemostatic effects have been achieved with transcatheter arterial embolization (TAE) for such perinatal bleeding, and there are reports of preservation of the uterus [1-8].

Embolic agents used in arterial embolization are divided into temporary embolic agents, such as gelatine sponges (GS), and permanent embolic agents, such as n-butyl cyanoacrylate (NBCA). NBCA has been used since the 1980s for conditions such as cerebral aneurysm, arteriovenous malformation, hepatic haemorrhage, and renal haemorrhage, and it has been reported to be an effective embolic agent. The mechanism of embolization with gelatine sponges and metal coils, which have conventionally been used in arterial embolisation, involves physically blocking blood flow and inducing thrombus formation around the embolic material. Therefore, in cases of severe perinatal haemorrhage with clotting abnormalities, there are risks of lack of sufficient embolization and haemostasis, as well as recanalisation due to recanalisation in a short period of time. In contrast, the mechanism of NBCA embolization involves a process in which liquid NBCA enters blood vessels, comes into contact with cations in the vessels, and polymerises, thereby becoming a solid substance that occludes the vascular lumen and achieves haemostasis. Although NBCA is used by mixing it with Lipiodol® (an iodised oil; Lpd), an oil-based contrast agent, the extent of embolisation can be adjusted by changing the mixing ratio. Specifically, increasing the proportion of Lipiodol results in prolongation of hardening time, enabling embolization in a template form to the periphery through the transfer of NBCA in the blood. In contrast, decreasing the proportion of Lipiodol shortens the hardening time and enables strong embolization of thick blood vessels as well. There are many reports of TAE with temporary embolic agents for massive bleeding in the peripartum period, after which pregnancy and delivery were possible [9,10], but there are no reports other than our previous reports [11,12] of pregnancy and delivery following TAE using permanent embolic agents for massive bleeding during the peripartum period. In our report, after the second delivery, CT angiography showed bilateral recanalisation of the uterine arteries that were embolized with NBCA at the first pregnancy. Thus, it is not clear whether NBCA is a temporary embolic agent. The mechanism of recanalisation of vessels embolized with NBCA requires further investigation.

In this study, the preservation of fertility with TAE using NBCA for massive haemorrhage during pregnancy or the peripartum period and the utility of this therapy were investigated.

## Material and Methods

Cases from 2005 to 2010 in which TAE was performed for massive haemorrhage in pregnant women, particularly during the peripartum period, were investigated. GS is generally used as an embolic agent in TAE, but in this investigation, NBCA was used with the following indications: (1) haemostasis was insufficient with GS; (2) there was severe DIC during the peripartum period; (3) obvious extravasation was seen with contrast CT or angiography. NBCA was combined with an oil-based contrast agent (Lipiodol^®^: TERUMO, Tokyo, Japan). Approval for the use of NBCA was obtained from the Clinical Trials Committee of the Institutional Review Board at St. Marianna University School of Medicine (approval number: 1034, NBCA-Lipiodol®  (NBCA-Lip) for use in cases of arterial bleeding accompanied by clotting dysfunction), and participants provided their written informed consent to participate in this study. NBCA was combined with an oil-based contrast agent (Lipiodol^®^: TERUMO Tokyo Japan). Statistical analysis was done using Student’s *t*-test and Mann-Whitney’s *U*-test, with P<0.05 as the level of significance. Data analysis was performed with the Statistics Package for Social Sciences (SPSS 12.0, Chicago, IL). 

## Results

From 2005 to 2010, 27 pregnant and peripartum women underwent TAE (Tables 1,2). The majority (14) had uterine atony. There were three with abruptio placentae, two each with caesarean scar pregnancy, placenta accreta, and vaginal laceration, and one each with cervical pregnancy, placenta praevia, pregnancy complicated with myomas, and amniotic fluid embolism. The embolic agent used was GS only in 5 cases, NBCA only in 19 cases, and additional embolization with NBCA when the effect with GS was insufficient in 3 cases (abruptio placentae, cervical pregnancy, and atonic haemorrhage).

**Table 1:** Details of 27 pregnant women who underwent TAE.

**Diagnosis**	**N**
**Uterine atony**	**12**
**Abruptio placentae**	**3**
**Placenta accrete**	**2**
**Vaginal laceration**	**2**
**Cesarean scar pregnancy**	**2**
**Pseudoaneurysm**	**2**
**Cervical pregnancy**	**1**
**Placenta previa**	**1**
**Pregnancy complicated with myomas**	**1**
**Amniotic fluid embolism**	**1**
**Total**	**27**

**Table 2:** All cases in which TAE was performed in pregnant women.

**No.**	**Diagnosis**	**Age**	**Gravida**	**Para**	**Gestational weeks**	**Blood loss**	**Blood transfusion (mL)**	**Agent**	**Hysterctomy**	**Pregnancy after TAE**
**1**	**Cesarean scar pregnancy**	**39**	**4**	**1**	**6**	**0**	**0**	**GS**		
**2**	**Abruptio placentae**	**33**	**0**	**0**	**35**	**Not recorded**	**28,120**	**GS**	**+**	
**3**	**Placenta accreta**	**36**	**1**	**1**	**37**	**1,824**	**1,200**	**GS**		
**4**	**Uterine atony**	**39**	**0**	**0**	**39**	**2,315**	**900**	**GS**		
**5**	**Uterine atony**	**34**	**0**	**0**	**40**	**2,227**	**1,320**	**GS**		**+**
**6**	**Cervical pregnancy**	**40**	**1**	**1**	**10**	**600**	**840**	**GS↓NBCA**		
**7**	**Abruptio placentae**	**35**	**2**	**0**	**32**	**4,750**	**10,190**	**GS↓NBCA**		**+**
**8**	**Uterine atony**	**27**	**1**	**0**	**40**	**2,875**	**37,940**	**GS↓NBCA**	**+**	
**9**	**Cesarean scar pregnancy**	**35**	**4**	**2**	**6**	**1,000**	**1,600**	**NBCA**		
**10**	**Abruptio placentae**	**29**	**0**	**0**	**31**	**1,439**	**9,320**	**NBCA**		**+**
**11**	**Placenta accreta**	**38**	**3**	**3**	**39**	**4,500**	**1,360**	**NBCA**		
**12**	**Placenta previa**	**42**	**3**	**2**	**37**	**4,790**	**8960**	**NBCA**		
**13**	**Pregnancy complicated with myomas**	**37**	**0**	**0**	**37**	**4,300**	**10,280**	**NBCA**		
**14**	**Vaginal laceration**	**39**	**0**	**0**	**41**	**3,296**	**10,220**	**NBCA**		
**15**	**Vaginal laceration**	**24**	**0**	**0**	**39**	**6,400**	**6,120**	**NBCA**		
**16**	**Pseudoaneurysm**	**36**	**1**	**1**	**32**	**Not recorded**	**0**	**NBCA**		
**17**	**Pseudoaneurysm**	**37**	**1**	**1**	**37**	**Not recorded**	**0**	**NBCA**		**+**
**18**	**Amniotic fluid embolism**	**44**	**4**	**2**	**40**	**8,820**	**25,240**	**NBCA**		
**19**	**Uterine atony**	**34**	**1**	**1**	**39**	**9,268**	**8,920**	**NBCA**		
**20**	**Uterine atony**	**29**	**1**	**1**	**37**	**3,965**	**18,660**	**NBCA**	**+**	
**21**	**Uterine atony**	**26**	**0**	**0**	**41**	**9,620**	**9,400**	**NBCA**		
**22**	**Uterine atony**	**36**	**4**	**0**	**39**	**4,530**	**3,960**	**NBCA**		
**23**	**Uterine atony**	**26**	**0**	**0**	**40**	**5,383**	**7,280**	**NBCA**		**+**
**24**	**Uterine atony**	**34**	**1**	**0**	**37**	**1,906**	**1,040**	**NBCA**		
**25**	**Uterine atony**	**37**	**1**	**1**	**38**	**7,868**	**9,760**	**NBCA**		
**26**	**Uterine atony**	**26**	**0**	**0**	**39**	**4,500**	**4,360**	**NBCA**		
**27**	**Uterine atony**	**41**	**7**	**5**	**38**	**3,304**	**3,320**	**NBCA**		

In No.2, No.16, and No.17, blood loss was unclear.

**Figure 1:**
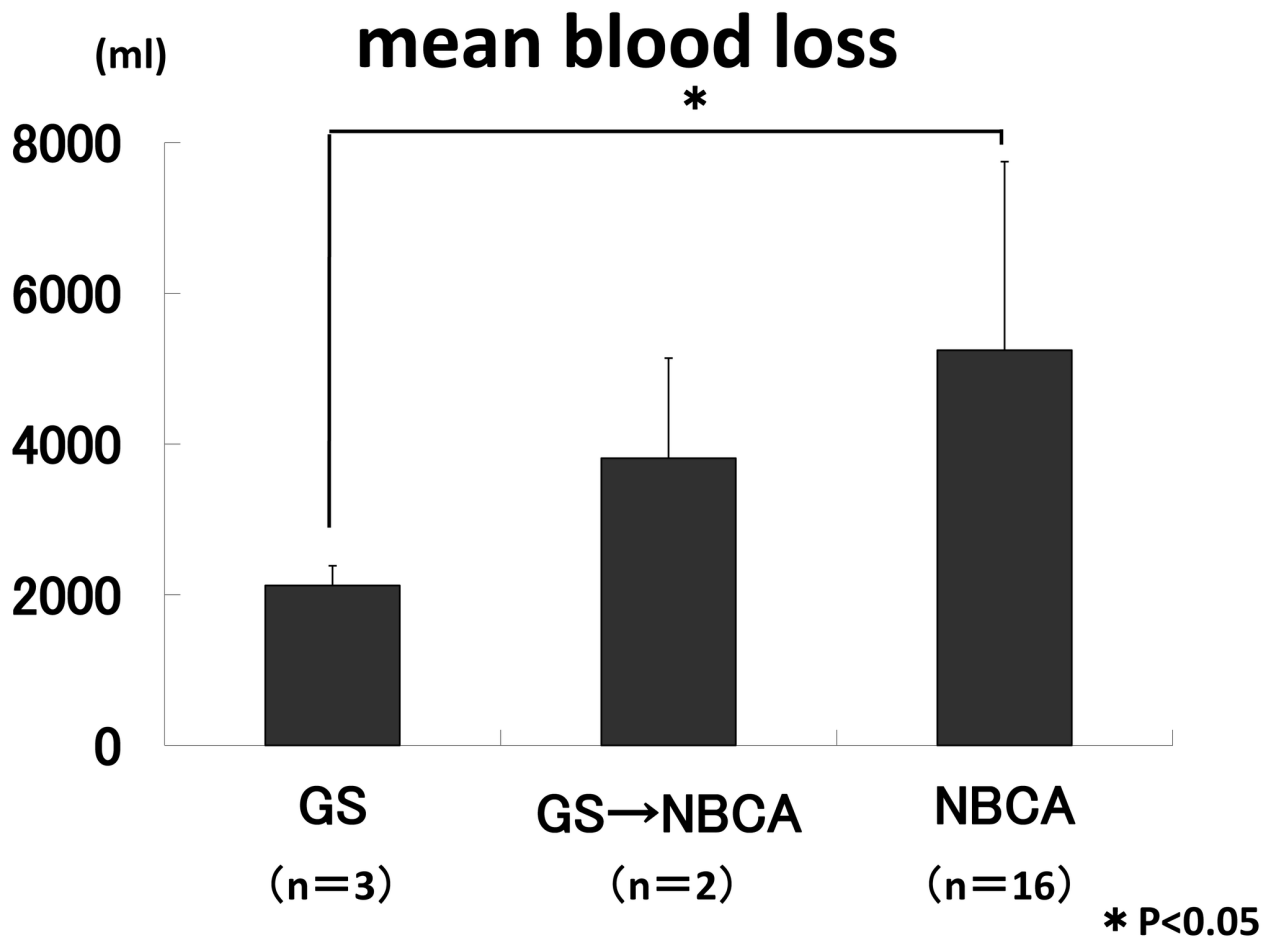
The mean blood loss with each of the embolic agents used for haemostasis was 2,122.0±261.8 mL with GS only, 3,812.5±1,325.8 mL with combined GS and NBCA, and 5,243.1±2,502.0 mL with NBCA only. A significant difference was seen between GS only and NBCA only (P<0.05) .

**Figure 2:**
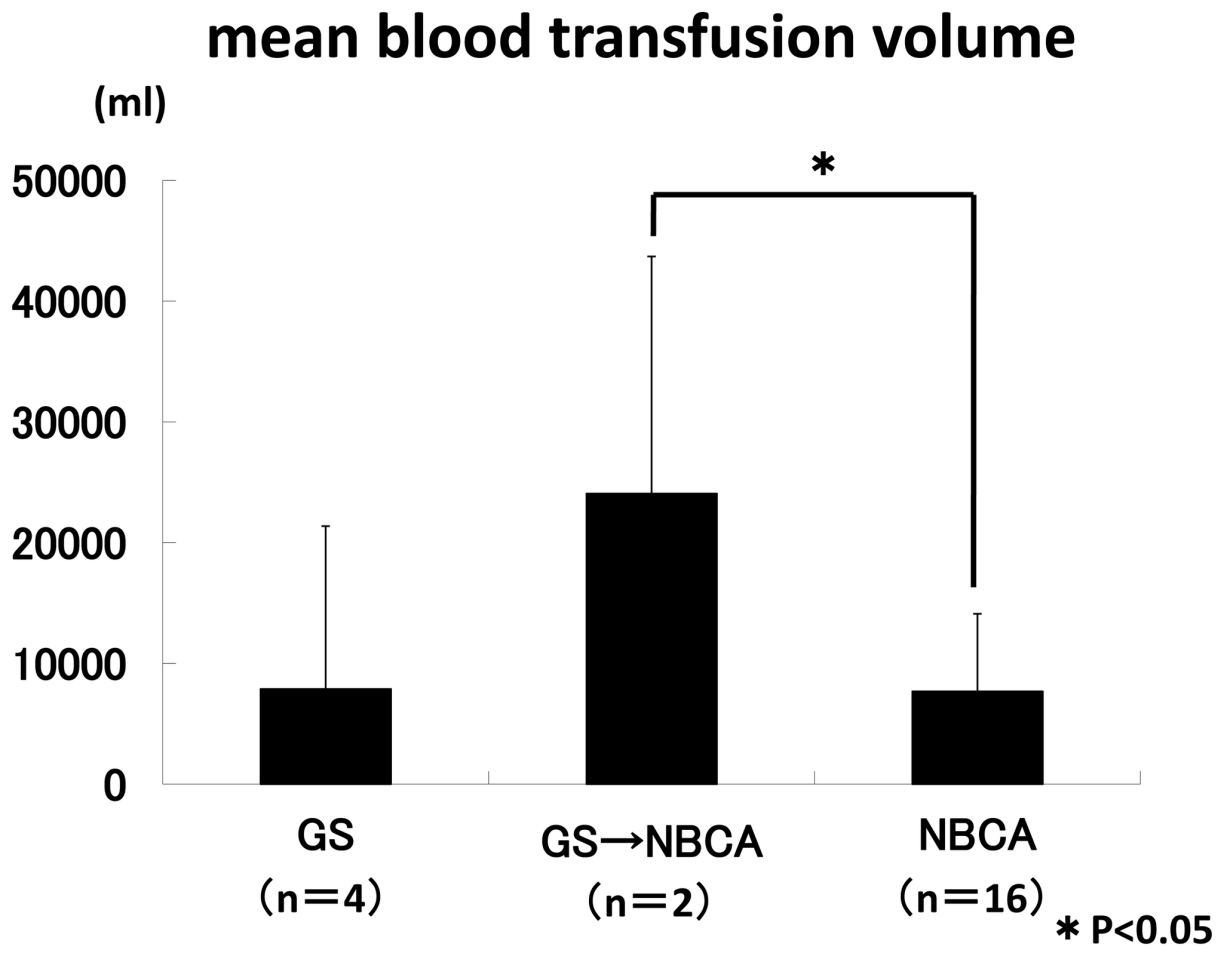
The mean blood transfusion volume was 7,885.0±13,491.2 mL, 24,065.0±19,622.2 mL, and 7,677.8±6,434.0 mL, respectively. A significant difference was seen between combined GS and NBCA and NBCA only (P<0.05).

Excluding the three cases that occurred in early pregnancy (one cervical pregnancy, two caesarean scar pregnancies), mean blood loss in the 24 patients who underwent TAE in the peripartum period was 4,661.0±2469.8 mL, and mean transfusion volume was 9,078.0±9,409.7 mL. The mean minimum Hb was 6.0±1.9 (0.9-9.3) g/dL, the mean minimum platelet count was 9.3±6.9 (1.8-31.8)× 10^3^/μL, and the mean minimum fibrinogen level was 132.8±94.4 (0-304) mg/dL. The mean blood loss with each of the embolic agents used for haemostasis was 2,122.0±261.8 mL with GS only, 3,812.5±1,325.8 mL with combined GS and NBCA, and 5,243.1±2,502.0 mL with NBCA only. A significant difference was seen between GS only and NBCA only (P<0.05) (Figure 1). The mean blood transfusion volume was 7,885.0±13,491.2 mL, 24,065.0±19,622.2 mL, and 7,677.8±6,434.0 mL, respectively. A significant difference was seen between combined GS and NBCA and NBCA only (P<0.05) (Figure 2). Excluding two cases in which NBCA was used in the peripartum period and the uterus was removed, menses had resumed in 8 of 18 patients at a postoperative follow-up survey. Four of them became pregnant, of whom three gave birth and one had a spontaneous abortion (Table 3). One patient had continuing amenorrhea.

**Table 3:** Course of pregnancy in patients who underwent NBCA during the peripartum period and later became pregnant.

**No.**	**Age**	**Progress**	**Gestational week**	**Mode of delivery**	**Neonate’s weight**
1	31	placenta previa, Pregnancy Induced Hypertension	32	Cesarean section	1804 g
2	37	Threatened Premature Delivery	37	Cesarean section	3131 g
3	27	Normal	37	Normal vaginal delivery	2640 g
4	38	cervical pregnancy			

## Discussion

NBCA is a tissue adhesive that is combined with Lipiodol^®^ (an oil-based contrast agent) and used as an immediate and permanent embolic agent. Its advantages are that: 1) the time until embolism and area of embolisation can be adjusted; and 2) the embolism effect occurs without dependence on clotting function. Its disadvantages are that: 1) the technique requires specific skills; 2) the risks of adverse effects from embolism or ischaemia, such as tissue necrosis, are similar to those with other embolic agents; and 3) in Japan, it is not covered by the national health insurance plan, and informed consent from patients is needed since, as a rule, its use within blood vessels is contraindicated. In practice, informed consent is first obtained preoperatively from the patient or, if the patient is unconscious, from the Obstetrics and Gynaecology Department and the Radiology Department.

**Figure 3:**
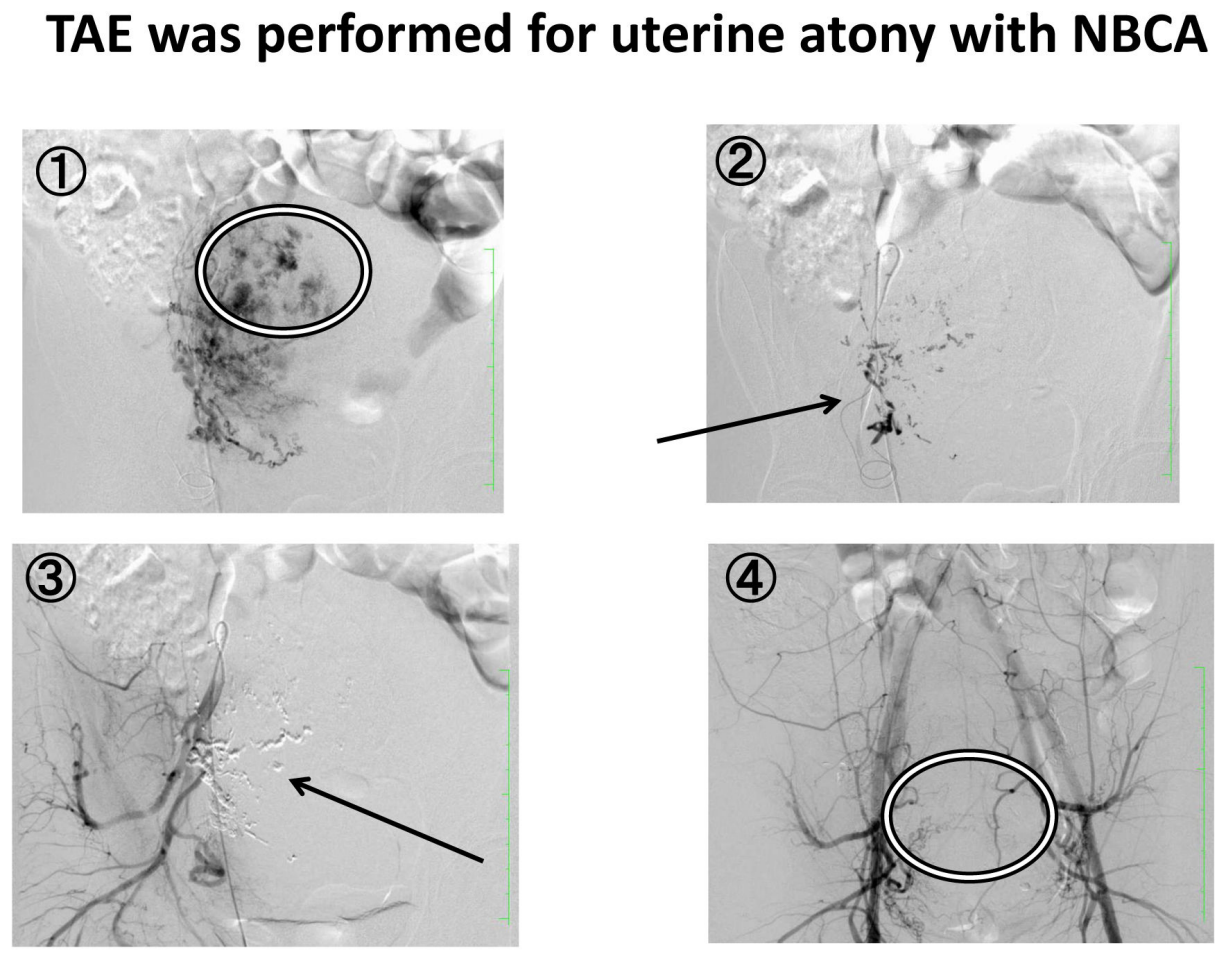
Uterine artery embolization with NBCA. (1) A catheter is inserted from the left or right femoral artery, arteriography of surrounding arteries is performed, and extravasation of the contrast medium is confirmed (inside circle). (2) The catheter is selectively advanced in arteries in which extravasation is confirmed, and the prepared NBCA mixture is injected. The flow of the injected NBCA mixture along the vessel could be confirmed while watching the contrast effect on perspective images (arrow) (3). The NBCA mixture hardens with time and embolises the site of bleeding. It can be visualised as a transparent solid substance on perspective images (arrow) (4). Contrast medium is used again after embolisation, but no obvious extravasation is seen, and reliable haemostasis has been achieved (inside circle).

In actual use, contrast medium (Lipiodol^®^) is mixed with 1 cc NBCA to improve visibility on fluoroscopic guidance. In addition, the mixture is prepared to match the vessel to be embolised, taking advantage of the fact that polymerisation time can be changed depending on the mixing ratio, and embolism is done under fluoroscopic guidance. With a 4-fold dilution, the polymerisation time is thought to be 4-11 sec. In the present cases, a catheter was inserted from the left or right femoral artery, arteriography of surrounding arteries was performed, and extravasation of the contrast medium was confirmed. The catheter was selectively advanced in arteries in which extravasation was confirmed, and the prepared NBCA mixture was injected. The flow of the injected NBCA mixture along the vessel could be confirmed while watching the contrast effect on perspective images. The NBCA mixture hardens with time and embolises the site of bleeding. It can be visualised as a transparent solid substance on perspective images. Contrast medium was used again after embolisation, but no obvious extravasation was seen, and reliable haemostasis was achieved (Figure 3).

Examination of 24 patients who underwent TAE during the peripartum period revealed a tendency for mean blood loss to be larger in the following order: patients with use of GS only, both GS and NBCA, and NBCA only. Blood loss was significantly greater in patients in whom NBCA only was used than in those in whom GS only was used (P<0.05) (Figure 1). Meanwhile, a comparison of the mean volume of blood actually used in transfusion showed nearly equivalent volumes in cases in which GS alone or NBCA alone was used, but significantly greater transfusion volumes were needed in cases in which both GS and NBCA were used than in cases with NBCA alone (P<0.05) (Figure 2). In other words, while the mean blood loss was higher in the NBCA only patients than in the other two groups, the amount of blood used in actual transfusion was little different from that in patients with GS alone.

The usage status and indications for embolic agents in TAE for massive haemorrhage during the peripartum period were as mentioned above. When clotting function was normal, GS was used in patients with little bleeding or in cases when DIC had not yet occurred, given that GS displays an embolic effect in cases when clotting function is normal. NBCA was used, and good results were obtained in patients transported to the hospital after massive haemorrhage occurred or in patients diagnosed with or suspected of having DIC from massive haemorrhage. These indication criteria are thought to be valid. In addition, among the cases given both GS and NBCA, there were patients in whom the embolic agent was switched to NBCA after embolization was first done with GS in patients with DIC, but good embolism was not formed because of abnormalities in clotting function. However, bleeding and a state of poor general control continued for a long time in these patients, and, as a result, a large transfusion volume was required. Currently, embolization is done using NBCA from the start in such cases.

The three patients who underwent hysterectomy were cases before the indication criteria were clearly determined. They were cases in which hysterectomy was performed first, and embolization was done as an adjunctive therapy to control postoperative DIC, or in which hysterectomy had to be done with non-proficient technique.

Complications with TAE are reported to be mainly infection or fever from embolism, skin or nerve disorders from ischemia, or necrosis of the uterus or bladder [13,14]. These are thought to result from embolisms that are formed in fine arteries other than the target site when GS is used in vessels from the internal iliac artery that are too fine, and from collateral vessels that do not function and supplement blood flow sufficiently. In our hospital, superselective arterial embolization is done by advancing a catheter to the uterine artery or internal pudendal artery and introducing the embolic agent, and we have not yet experienced these complications. The only complication that occurred was in a patient with embolization done following hysterectomy in whom bleeding from a vein was mistakenly judged to be bleeding from an artery. Extravasation was seen in a nearby artery, the mesenteric artery, but embolization was done, resulting in necrosis of the intestinal tract, and emergency resection and a temporary colostomy were performed. During the laparotomy for the bowel resection, the extravasation was found to be in the left ovarian vein, which was thought to have been damaged during the hysterectomy (Table 2, No 8).

## Conclusions

TAE was performed for massive bleeding during the peripartum period, after which menstruation was restored, and five of the patients became pregnant. GS was used in one of these patients, NBCA was used in four, and babies were delivered by three. TAE using NBCA is safe in patients with massive haemorrhage during the peripartum period and can preserve a uterus capable of pregnancy, thus maintaining fertility.
